# Exploring pandemic preparedness in higher education: lessons learnt from students’ lived experiences during a critical time

**DOI:** 10.1007/s44250-023-00024-y

**Published:** 2023-03-02

**Authors:** Jinan Abi Jumaa, Rodolfo Catena, Elliot Brown, Saikou Sanyang, Alessandro Tridico, Dawn Weaver

**Affiliations:** 1 School of Health and Care Management, Arden University, Dessauer Str. 3-5, 10963 Berlin, Germany; 2 UCL Global Business School for Health, Gower St, London, WC1E 6BT UK; 3 School of Health and Care Management, Arden University, Middlemarch Park, Coventry, CV3 4FJ UK

## Abstract

**Background:**

The COVID-19 pandemic has had a marked impact on educational disruption and progression of students. Linked to this, studies have demonstrated increases in depression, anxiety, and stress, with long-term outcomes yet to be understood. Students in Higher Education (HE) were at particular risk due to circumstances such as financial stress from job loss, shifting to online learning and uncertainties about the future, with many international students isolated from social support networks. This study explored lived experiences of determinants for academic disruption in HE students during the COVID-19 pandemic across Germany and the UK.

**Methods:**

The study used qualitative secondary data collected from extension and mitigation claim forms from 2019 until 2021 from a university with campuses in the UK and Germany. A phenomenological perspective was utilised to draw out experiences and insights into determinants for mitigation from students to enhance our understanding of real problems encountered during a period of crisis. Thematic data analysis was used to create themes of influence for mitigation of assessments.

**Results:**

Themes identified pre and during the COVID-19 pandemic included; pre-COVID: work-related commitments; bereavement; illness of a family member; mental and physical health issues; natural disasters, during 2020/21, themes created were; COVID-19 social impacts; workplace and financial demands; psychological distress; physical illness, with subthemes evolving such as family responsibilities; and caring for others; furlough and its financial impacts; heavy workload for frontline health care workers; mental health impacts; physical abuse and crime, COVID-19 physical symptoms.

**Conclusion:**

We suggest an Integrated ‘Determinants of Wellbeing Framework’ for supporting HE students during critical times such as a pandemic. Our suggested framework was adapted from determining health inequalities and the concept of the ‘flourishing student’ that maps the relationship between the student, their environment and well-being. It is hoped the framework will serve to inform future theories around disruption to student progression and to explore the relevant impact on educational outcomes in HE thus assisting in appropriate support planning.

## Introduction

During the critical time of COVID-19, a vast proportion of initial research justifiably concentrated on the epidemiology, clinical studies, transmission and treatment of the virus [12, 21, 26]. However, several studies have considered the effect on the mental health and well-being of university students and the influences associated with higher levels of distress [15, 24]. These studies have added to our understanding of the effects of a crisis, such as the pandemic, on students striving to achieve academically. To further our interest in critical events and disruption for HE students, this study explored the experiences of individuals as they wrote them in their applications for mitigation and any risk factors impacting their ability to submit their assessments to meet the deadlines, during this pivotal time. The authors wanted to draw attention to the lived experiences of their Higher Education (HE) students who are not traditional students but rather mature ones having work and other family commitments, explaining the context of mitigation cases before, during and after lockdown periods of assessment submission. This would help reveal the pressures and challenges faced by students during a critical period such as the COVID-19 pandemic.

To add context to this critical time endured, severe acute respiratory syndrome coronavirus 2 (SARS-CoV-2), the seventh human coronavirus, was first discovered in Wuhan, China [20]. Its discovery unfortunately led to the rapid enforcement of restrictions in societies globally. The restrictions for some, eventually turned into a full lockdown to contain the virus. Such communities understandably faced regional lockdowns to starve the pandemic of its harmful outcomes, but in its tracks, it impacted the vulnerabilities of individuals. In line with this, to further decrease the spread of the virus, educational institutions closed, transitioning to online learning [14].

### Aim

The study aimed to create awareness of the interconnectedness of a critical event and the many factors faced by HE students in the United Kingdom and Germany. Students lived experiences during periods of assessment deadlines provided a ‘voice’ for social circumstances, physical illness and psychological distress encountered during this critical period.

### Objectives

To evaluate and understand student experiences that impacted their ability to achieve assessment deadlines during the critical period of the COVID-19 pandemic.

To develop a framework to provide a further understanding of the need for support to address the determinants for mitigation during critical times.

To make recommendations for policy intervention and integration of services to address HE student resilience during critical times.

## Literature review

Embedded in this study is the underpinning model of the ‘flourishing student’ [23] and it forms a basis to conceptualise the notion of the very pressures on higher education students in their journey of university life and how they can be counteracted. In Higher Education Institutions, the mission revolves around the idea to nurture graduates who can succeed, and this means nurturing the student’s well-being, thus supporting the concept of the ‘flourishing student.’ Vailes [23] draws on Bernard’s [1] definition of resilience, which encapsulates resilience as an innate ability, impacted by environmental influences which must demonstrate protectiveness. To deviate from this concept leaves students, open to issues of vulnerability and mental ill health. Several studies that follow indicate the deviation from the ‘flourishing student’ model during a critical time.

A recent study of one UK University [4] recognised high levels of anxiety and depression in undergraduate and postgraduate students early in the COVID-19 pandemic in England and relatively low levels of resilience. Interestingly, they comment that: ‘given the global context for these increased levels of psychological distress…. it is important to see this as an understandable reaction to an adverse situation which may be transitory’ (p. 15). Chen and Lucock [4] in arguing for the type of support students would need in future, worryingly acknowledged there is a prospect of the emergence of a significant number of students developing long-term difficulties with potential physical and mental health connotations. This is an important element here in that we need a continuum of support interventions to ensure students can develop confidence in maintaining their mental health and well-being.

A survey of undergraduate students by the Higher Education Policy Institute (HEPI) asked students how the COVID-19 pandemic had affected their mental health. Of these, 58% said their mental health had become worse, 14% said it is better, and 28% said it was the same [11]. Correspondingly, the Coronavirus Student Survey phase 3, a combined survey for both HE and Further Education steered by the National Union of Students (NUS) [16]. ascertained that 52% of students expressed their current mental health and well-being as worse (35% described it as the same, 8% as better, 4% refused, in comparison to pre-pandemic life. Notably, they report that 3 in 5 students had not pursued support, possibly signifying an absence of awareness or accessibility of help.

To extend this consideration of pertinent statistics during the critical time of COVID-19, Defeyter et al. [7] noted with interest how housing worries, food insecurity, social distancing—linked to social isolation, and the competing challenges of family responsibilities had further worsened the abilities of HE students to flourish [7]. Collectively, these issues perpetuate inequalities (e.g., social inequalities and diminished multistakeholder collaboration [19] especially in the promotion and achievement of educational outcomes). This is something key stakeholders could generally work on to improve collaboration across sectors, thereby safeguarding the ‘student voice’ and ensuring its role remains central in decision-making about student well-being.

The researchers, having worked through the pandemic supporting HE students themselves wish to provide their autobiography as practitioners who played an active role in implementing policies to support students and institutional capability. It is from this positionality we argue that our role as academics and academic managers is particularly important in surfacing the disruptive factors faced by students during a critical period between 2020 and 2021. Indeed, there is a need to raise awareness about not just the physical impact experienced by HE students at the time but that the pandemic had exerted incredible power on students’ lives, requiring them to stay in quarantine, change daily routines, suffer the loss of employment and increased financial pressures [22].

Stress and coping are useful concepts in conceptualising applications for mitigation, particularly concerning the experience of a ‘crisis’ of ‘critical events’, broadly coping mechanisms are based upon ‘approach’ and avoidance, meaning facing an event or moving away from it. Our findings embrace this while at the same time attempting to explain how we can move towards understanding and anticipating support and provision for student services in HE. For example, within a conceptual framework that pays attention to social situatedness in a way, we could anticipate, plan and mitigate future stressful threats and proactively support student progression. This corroborates [22] who argued that post-pandemic there is an urgent need for more interventions and preventive mechanisms to be in place to ensure HE students’ well-being is prioritised.

To capture our research, we drew on the model of Dahlgren and Whitehead [6] as it has demonstrated connections between the individual, their environment and health. It utilises the underpinning of a biopsychosocial perspective to develop a framework to address academic disruption in a crisis, we further integrate the concept of the ‘flourishing student’ to enhance our understanding of the determinants and impacts of the crisis on students.

## Methodology

Qualitative secondary data collected from extension and mitigation claim forms were the source of data for this pilot study. Data from 2019 until 2021 from a UK university with campuses in the UK and Germany were analysed following approval by the Ethics Review Committee of the education institution. Data included the following variables: Ethnicity, Gender, Age, Disability, Nationality, Country, Study Mode, Study Centre Name, Mitigation Status and Mitigation Reason, Board Decision and Student Attendance Percentage. Our research interest was to look at the ‘lived experiences’ documented in the mitigating reasons to ascertain determinants during the submission of assessments to meet deadlines. Data was made available to researchers under the Data Protection Act (2018) and anonymised before our receipt for analysis. The UK university provides higher education through part-time online distance learning and full-time blended learning through its Study Centers. Its mission is to remove the barriers to higher education, inspire new ways to learn and enrich people and their lives. The vision is to be known as the University that made higher education more accessible and beneficial to all.

### Qualitative analysis

Qualitative data constituted of answers on the mitigation applications to the question “*What is the reason for applying for mitigation?”* As researchers, we introduced our own reflexivity here and worked with our insights and experiences as researchers and lecturers to help us understand the context of our research and acknowledge our subjectivity. We firmly acknowledge our position formed part of the process and has some impact on that process, and it is considered part of the research and its reporting. A constructionist approach was utilised for the thematic analysis as the data retrieved was to form the basis for a framework of determinants for factors disruptive to student progression. The data thus was analysed to capture the biopsychosocial elements of a “critical time”. A latent approach to thematic analysis allowed us to look at the data and meanings at a much deeper level and vindicated with a theoretical framework. Due to our own experiences during the pandemic period and consideration of previous research, we acknowledged some preconceived themes would occur.

Three researchers conducted the thematic analysis to extract ‘*shared meanings and experiences*’ ‘that inhibited students from meeting submission deadlines [2]. All data that was generated from the mitigation claims were coded and analysed by three researchers in the team that met regularly to prevent bias in the results and discuss the validity of the codes and categories [5]. The coding of mitigation reasons was done manually using an inductive approach. Even though the process was mainly iterative and time intensive, we felt this was necessary to have an unprejudiced view of the themes—that is, to ensure we best represent meaning as it is captured in the mitigation information from students [3].

**Fig. 1:**
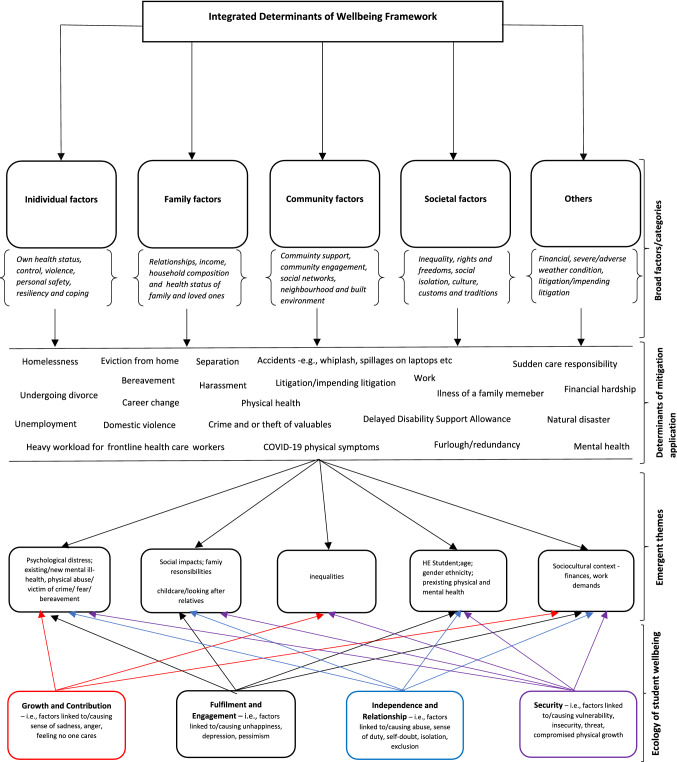
Determinants impeding student progression during critical times (2022); adapted from Dahlgren and Whitehead [6] and Vailes [23]

There was a realisation of theoretical saturation as common themes and subthemes were revealed, and there was adequate commonality. The addition of the researchers’ own experiences in teaching during the pandemic period reflected our representations of the students’ mitigation reasons [9]. Figure 1 depicts the resulting framework and the conception of themes from student voices.

## Results

To add context to our sample; 34,855 students applied for mitigation in 2019 (21,502 females and 13,348 males), and this increased to 48,737 in 2020 (29,167 females and 19,559 males. Distance Learning (DL) showed the highest number of mitigation applications across both genders and both years. Tables 1 and 2 give a summary of the Sociodemographic Characteristics of the Study Population in 2019 and 2020.

**Table 1:** Sociodemographic data 2019

Variable			
Gender			
F	21,502		
M	13,348		
O	4		

	Average of age	Max	Min
Age			
F	35,15	80	20
M	35,75	85	19
O	30,25	31	28
Location			
Germany	1544		
UK	18,411		
Distant	14,709		
Degree apprenticeship	190

**Table 2:** Sociodemographic data 202

Variable			
Gender			
F	29,167		
M	19,559		
O	10		

	Average of age	Max	Min
Age			
F	33,68	80	19
M	33,58	83	19
O	29,20	31	28
Location			
Germany	4519		
UK	26,314		
Distant	17,518		
Degree apprenticeship	385		

Six themes were created in the mitigation claims of 2019 as described below:

### Work-related commitment

The students we have in our sample are not traditional students who have just graduated from high school, but rather mature students who have work commitments in health and social care roles. The complex set of decisions that these students must make includes deciding how to pay for their degree, how much they need to work to cover the fees, how many jobs to take, how to manage their family and children, and how to balance these conflicting priorities while pursuing a degree.

*“Early June 2019 I lost my job. This was an unforeseen circumstance had to dedicate a lot of my time and energy looking for a new employment. I began the module positively, however my concentration and attention were strained, and I went through a stressful time”.*

### Bereavement

Students who have experienced a death of a family member are among those who are most at risk for continuing mental health issues in higher education. In our study bereavement was a recurring theme that was experienced by many of the students and was the main reason for applying for mitigation.

*“I have been suffering with the death of a close family member who played an active role in me and my daughter’s life. This has a significant impact on my mental health. My migraines have come back a lot worse than usual, stress and anxiety are also what I have been suffering with.”*

### Illness of a family member

The illness of a family member was also a repetitive theme in our study, most of the students who applied for mitigation were the main caregivers of their families. Due to their caring responsibilities, these learners experienced substantial pressure, stress, and depression. While caring for a loved one and at the time having to fulfil their university demands caused a lot of stress and required them to apply for mitigation. One student, for example, mentioned that she was unable to complete the assignment as she was looking after her mother who had cancer.

*“Due to the coronavirus I have had no childcare for my children, one of which is disabled, this has left me with very little time to do any of my work or watch any of the online lessons. Usually I am able to get childcare for my children if I am struggling on time to help me get my work done”*

### Mental and physical health issues

Mental health which is related to all the above-mentioned themes was one of the main reasons students were experiencing difficulties in their studies, not being able to submit their assignments on time, and thus applying for mitigation.

Depression, stress, and anxiety were the main mental health conditions mentioned.

*“I was depressed after being in debt for a long period of time- I have used my student loan to pay debts and I currently cannot afford to buy food; I am living on biscuits”*

Mental health as observed in the above quote, we found interlinks with other themes explored in the section. For example, it could be brought on by the illness of a family member, bereavement, work-related commitments, and financial difficulty which we have categorised below under other personal reasons. In addition to mental health, physical health was also a recurrent theme. One student mentioned that they had been suffering from a severe cough. They were medically assessed and diagnosed with viral respiratory tract infection and thus unable to concentrate and lost a significant amount of time from studies.

### Natural disaster

While this appeared to be mostly cited as a reason for mitigation by distance learning students in comparison to blended learning, we noted that Hurricane Dorian hit the Bahamas in early September 2019. The effects of this hurricane were among the greatest that inhabitants of the Island had ever seen. Some of our students were in New Providence, Bahamas and were affected by the hurricane. Other students were required to work extended hours at emergency shelters to help people affected.

Hurricanes such as Irma, Maria, and Dorian also impacted the British Virgin Islands (B.V.I.). The pre- and post-effects of the hurricanes resulted in further damage to infrastructure and presented added challenges such as power shortages and outages, limited and or poor internet access, flooding and water damage issues, thus negatively impacting student performance.

### Other personal reasons

Lawsuits, stolen property including laptops, financial issues and issues with landlords were frequently reported as reasons for applying for mitigation.

One female student also shared that she had been involved in a domestic violence incident with her ex-partner who physically assaulted her and kept her locked out of her home. She explained her mobile phone was also seized by her ex-partner and was only able to retrieve this with the help of the police.

Table 3 below describes the main themes and subthemes for reasons for applying for a mitigation claim in 2020.

**Table 3:** Themes extracted from the qualitative data of student mitigation claims (2020)

Themes	Sub-themes
COVID-19 social impacts	• Family responsibilities and caring for others
Workplace and financial demands	• Furlough and its financial impacts • Heavy workload for frontline workers
Psychological distress	• Mental health impacts • Physical abuse and crime
Physical illness	• COVID-19 physical symptoms

### COVID-19 social impacts

In 2020, a large proportion of students struggled to maintain a work-life balance during the lockdown. They had to take care of children, loved ones and other personal responsibilities while at the same time studying or working from home. This resulted in a lot of stress. In line with this theme, two of the participants explained:

*“Due to COVID-19 I have been working at home and as a single parent I have been solely looking after my nine-year-old daughter since 20 March due to the lockdown.”*

*“Due to the current pandemic. My circumstances have changed. I have two children age 9 and 3 and they are both at home full time. I have not been in a position to study during the day. I have tried to manage my study during the evening, but there has just not been enough time.”*

### Workplace and financial demands

This was the most significant theme in the data set of 2020. Beyond the physical and mental health effects of the pandemic, the reduction in economic activity resulted in a fall in employment and income, and students lost their jobs which resulted in financial and mental health pressures. This was a recurring theme throughout the data.

*“I have recently been made redundant from my current job and am trying to find another organisation who can take over the sponsorship of my degree apprenticeship”*

On the other hand, many students working as front-line workers during the pivotal period of the pandemic faced increased workloads which negatively affected their studies.

*“I am key worker and I work nights at a fairly large care home. During this pandemic I have been working non-stop working due to shortage of staff and it has consequently affected my health. I have been exposed to many residents who have tested positive for COVID-19. I am currently isolating, and I am on the sick at work. My symptoms, which have just started to appear, resemble those of COVID-19. Due to the shortness of breath and lethargy/fatigue, I have been unable to complete the rest of my assignment”*

### Psychological distress

The COVID-19 pandemic has caused a lot of pressure on the mental health of students. Many reported experiencing stress. Fear of catching a disease, long working hours, unavailability of protective gear and supplies, patient caseload, unavailability of effective COVID‐19 medication, death of their colleagues after exposure to COVID‐19, social distancing and isolation from their family and friends, and the dire situation of their patients may take a negative toll of the mental health of health workers.

*“Recently my close family member has passed away from COVID-19. This is horrible for me and I can’t express how I am feeling. I have also been stressing about my dissertation and the due date is near, which has put more anxiety on me. I was hoping to get more time to complete my research project.”*

Physical abuse was also reported during the lockdown among some students, one female student mentioned that she had to flee from domestic violence and stayed in a women’s refuge and thus was not able to concentrate on her studies during this critical time.

### Physical illness

While not exclusively in all cases, we did observe that physical illness was mainly attributed to some of the physical symptoms of COVID-19. For instance, fever, muscle pain, feeling exhausted, cough and diarrhoea. For these students, we noted the advice from the authorities to self-isolate negatively impacted their well-being.

For example, one of the students said:

*“I belong to the vulnerable group due to my diabetes. I am having high temperature and fever and occasional tiredness however this is very unusual for me in normal times, I can only relate it to symptoms of corona virus. I am able to isolate and manage the symptoms but the fear particular is something I could not overcome this is the reason I could not concentrate”*

### Proposed framework

We developed our framework from both Vailes [23], who directed her thoughts to the preservation of the ‘flourishing student’ aligning them with the mission values of the University under study, and Dahlgren and Whitehead [6], who identified circumstances that govern the quality of the health of the population. From this, we were inspired to demonstrate the interconnectedness of the circumstances that determine the disruption to the academic progression of HE students and the determinants that influenced their decision to mitigate their assessment deadlines during critical times.

The framework represents a model of the numerous factors from the findings that interact and has policy connotations for partnership working and further integration of services between health and education, prompting HE educationalists, into initiating actions and interventions that may counteract these determinants to provide preparedness for future critical times.

The framework reflects the HE students’ experiences from diverse cohorts during a critical time in their lives and captures priority areas for action. Physical and mental health are to be seen as closely interconnected and affect each other through several pathways, which is represented here and underpinned by a biopsychosocial perspective, in which health is identified as being an artefact of biological, psychological, and social processes. The framework paves a way to link the themes to potential service categories to provide solutions or preventative measures from a range of different services to address or pre-empt personal crisis states emerging.

## Discussion

The COVID pandemic after its outbreak in China in 2019, rapidly spread across different countries. Such was the devastating impact that WHO declared a public health emergency of significant global concern in March 2020 [8].

This caught a lot of HE providers by surprise and resulted in the suspension of face-to-face teaching activities. This was swiftly followed by the switch to online or virtual learning. Although this was viewed as a crucial strategy to mitigate the disruption to learning, it could not have possibly foretold or mitigated the untoward impact it would have on students [13].

Furthermore, to mitigate concerns students, governments and industry had about well-being, equity of access, quality, and assessment fairness given the pandemic, our HEI as well as many others in the UK introduced a ‘No Detriment Policy’, the purpose of which was to ensure no student is academically disadvantaged by the changes brought on by the pandemic. The Quality Assurance Agency (QAA), in their thematic guidance published in early April 2020 stated that many providers were right in “…introducing ‘no detriment’ or safety net models in response to the coronavirus pandemic; and those who had not yet announced such policies were coming under pressure from student bodies and others to do so.” [18].

Despite the no-detriment policy and other support provided for HE students, our findings suggest that a lot of students continued to experience the impact pandemic on their mental and physical health.

We have explored, using the Covid -19 pandemic as an illustration of a critical period why academic disruption has led some students to apply for mitigation, a request to postpone the assessment of their courses. We have created the following main themes from our data, social impact, workplace and financial demands, psychological distress and physical illness. Reasons to apply for mitigation included the impact of the pandemic on students’ financial needs and their caring responsibilities. Significant changes to daily life; the risk of temporary unemployment; home-schooling of children; caring for others and lack of physical contact with other family members, friends, and colleagues; were all recognised explanations for mitigation.

The integration of the ‘Flourishing student model’ [23] and the establishment of our own proposed framework from our findings has served to enable theoretical generalisability, which resonated with our own reflective experiences during a critical time as lecturers. Future preparedness is our recommendation for maintaining the ‘flourishing student’ and combat the reduced resilience in future periods of crisis. We would like to extend our recommendations to the agenda of integrated working between health and education to address the working nature of HE institutions and its support of students and staff in conjunction with community-based health provision.

The pandemic has had a significant impact on female students. COVID-19 has had the greatest impact on employment opportunities in sectors that are dominated by women affecting the lives of the students who work and study at the same time. This seems to confirm with Prowse et al. [17] who posited that female students appeared to be disproportionately affected by the negative impact of the pandemic when compared to males. Even so, our sample included only health and social care students and there is a realisation that mitigation requests may have reflected the increased workload for front-facing staff. In general, the themes we have created during the pandemic reflect the themes we identified before Covid-19. This finding implies driving partnerships in the management of health systems and reducing the risk of medicalising social issues, which may impact the accessibility of community-based services. If health systems had developed policies to tackle these challenges effectively before the pandemic, the impact of Covid-19 on academic disruption would have probably been more limited.

As researchers, we realise the limitations of our research, as the sample is from one UK university and of healthcare management students, some of whom were key workers who may have been disproportionately affected by the ‘workload element’ increase and ‘exposure’ to ‘Covid-19’ resulting in physical illness in the claims for mitigation. Future research needs to be done to improve acknowledgement in different universities around the world including LMICs. We also acknowledge that nuances in the data may have been missed, and our increased reflexivity has allowed us to reflect carefully on choices and interpretations.

The existence of data from two different countries is another limitation to consider and further research will need to shed light on how ‘country of residence’ moderates the impact of the determinants we have identified. The United Kingdom and Germany adopted different strategies to support their populations that may have reduced or increased the impact of the determinants. For example, the pay for sick leave in the United Kingdom is one of the lowest among OECD countries [10]. This aspect can further exacerbate the impact of financial demands. Mitigation forms may capture only the reasons students are willing to share with individuals external to their families. We assume that students can express and articulate all the reasons causing them to delay their education. Consequently, unconscious factors may have been neglected in our analysis. Continuation of future research will need to consider how to capture this additional insight.

Opportunities for future work will also need to assess the relative impact of the determinants we have identified and clearly illustrate how they interact together. As these students are part of the future health systems workforce, we need to determine which policies to reduce the disruption of education we need to prioritise. Future analyses will need to evaluate the impact of these determinants on societal costs. For example, how psychological distress becomes a burden for the whole society and why preventive policies may have a positive financial return if they reduce this burden.

## Conclusion

The Covid-19 pandemic has put to the test our education system and illustrated the digression of the ‘flourishing student’. We have explored and exposed the ‘voices’ of health and care management students that highlight some of the possible determinants of why this pandemic has disrupted education. It is important to convey the lived experience of students that caused them to mitigate their assessment during the pandemic and consider scaffolding mechanisms to support students in the event of future outbreaks or critical times.

The Inclusion Team at the university is committed to reducing barriers to students’ studies and providing the best possible support. In order to make sure students have the proper support in place and can access the resources they need; they created a hub online for reducing barriers to study to reduce academic pressure and signposting relevant charities and support groups. This hub includes free online welfare advice, as well as well-being support 24 h a day, 365 days a year. The university has worked to reduce students’ avoidant coping strategies through support services, including psychological support. The determinants described in this study demonstrate that there are continuing opportunities to redesign support mechanisms in ways that could improve educational outcomes and progression for HE students. Future work needs to be done to improve our management of academic disruption. During periods of critical events, we ground our provision in improving familiarity around mental health issues and stress in academia and developing emotional resilience and mental well-being among students. To conclude, the WHO predicts a shortage of 15 million health workers by 2030 [25], thus improving educational outcomes in pandemic and non-pandemic critical event contexts and the maintenance of the ‘flourishing student’ becomes an essential requirement for education and health systems worldwide.

## Data Availability

The data that support the findings of this study are available from the corresponding author, JAJ, upon reasonable request.
